# Sex Differences in Perception of Economic and Dating Access

**DOI:** 10.1177/14747049241310154

**Published:** 2025-02-09

**Authors:** Rachel E. Hall, Khandis Blake, Ho Fai Chan, Benno Torgler, Stephen Whyte

**Affiliations:** 1 School of Economics and Finance, 1969 Queensland University of Technology, Brisbane, Australia; 2 Centre for Behavioural Economics, Society & Technology (BEST), 1969 Queensland University of Technology, Brisbane, Australia; 3 Melbourne School of Psychological Sciences, 2281 University of Melbourne, Melbourne, Australia; 4 CREMA—Center for Research in Economics, Management, and the Arts, Zürich, Switzerland

**Keywords:** sex difference, gender, online dating, mating market, labor market

## Abstract

Mating and labor markets are fundamental drivers of societal dynamics. Yet, equity of access to these domains differs between the sexes due to numerous biological, economic, psychological, and socio-cultural factors. These inequalities and their impacts can accentuate perceptions, preferences, and behaviors of males and females in different ways. Utilizing a large cross-sectional sample of those currently engaged in the Australian mating market (*n *= 1072 online daters), we explored the impact of sex and individual differences on the perceptions of men's ease of access to a decent job in the labor market (opportunity), women's economic dependence on men (economic inequality), and relative reproductive opportunity (dating access) for both sexes. Our study identifies both sex differences and symmetries in socio-economic factors (such as education level and having offspring) correlating with the perceptions of both economic and dating market access for Australian online daters. Additionally, key resource endowment indicators (income and unemployment) also reflect differences in both sexes’ perceptions of both access and gender equity. That said, our study finds that perceptions of access to both (economic and mating) markets shows far greater variation based on biological age (especially for women) than any other factor.

## Introduction

Two prominent theories of human mating offer different explanations for reproductive decisions and behavior. On the one hand, social constructionist theories of sexuality explain differences in individuals’ preferences and decision making to be the product of specific cultural, social, and patriarchal forces (Brickell, 2006). In contrast, evolutionary and sociobiological theories attribute modern sexuality and resulting behaviors to evolved context dependent adaptations, which maximize opportunities to pass on one's genes (Buss & Schmitt, 1993). This is not to say that either perspective believes nature or culture to be the sole explanation for mate choice decision-making and behavior, but rather they differ in their understanding of the relative contribution of each (Baumeister, 2000). Nevertheless, DeLamater and Hyde (1998) argue that the two views have fundamentally opposed underlying assumptions. That is, while the evolutionary approach relies on underlying “true” preferences, discontinuity between forms, and consistency across time and cultures, social constructionists assume the opposite (DeLamater & Hyde, 1998). Indeed, patterns of homogamy and sex^1^ differences in mate preferences are evident across different cultures (Buss, 1985; 1989; Walter et al., 2020), challenging the social constructionist view. However, recent research highlights discrepancies between societal perceptions and individual attitudes, particularly regarding sexual double standards. For instance, Kennair et al. (2023) found that while individuals often perceive sexual double standards to exist at a societal level, their own attitudes and behaviors frequently deviate from these norms, revealing a complex interplay between perception and practice. On the other hand, while the evolutionary approach identifies universal preferences for physical attractiveness which are believed to maximize fitness, social constructionists highlight cultural variations such as shifting beauty standards across time (DeLamater & Hyde, 1998).

Sexual Economics Theory (SET) has also emerged, which recognizes that marital patterns vary across time and cultures, like the constructionist view, and claims that such shifts in reproductive behavior can be explained by understanding marriage as a “market” which can be modeled according to utility and equilibrium assumptions (Baumeister & Vohs, 2004; Becker, 1973). SET has been extended by applying the economic principle of exchange to the sexual marketplace, where women are “sellers” while men are “buyers” (Baumeister et al., 2017; Baumeister & Vohs, 2004; Whyte, Brooks, et al., 2019). To gain access to sex, men must offer women sufficient value in terms of nonsexual resources, and this “price” is influenced by numerous individual and market factors (Baumeister & Vohs, 2004). For example, a larger pool of men than women raise the “price” as demand exceeds supply (Baumeister & Vohs, 2004). All these theories of mate choice agree that both men and (especially) women's sexual behavior can be highly responsive to cultural, social, economic, or ecological changes (Baumeister, 2000; Becker, 1973).

Economic inequality is one such market condition producing observable shifts in women's reproductive strategies (Barber, 2001; Blake et al., 2018; Brooks et al., 2011; Loughran, 2002). Long-term mating has typically served as a vehicle for women to gain resources, either to improve their own wealth and status (according to cultural or economic explanations), or to enhance the survival of their offspring (Baumeister & Twenge, 2002; Baumeister & Vohs, 2004; Blake & Brooks, 2019; Wiederman & Allgeier, 1992). Economic inequality not only influences resource availability, but also shifts reproductive strategies, amplifying hypergamous tendencies by increasing women's preference for partners with higher socio-economic status (Blake & Brooks, 2019). This effect can disproportionately affect lower-income men and higher-income women, creating disparities in mating success. Furthermore, men in economically disadvantaged positions may perceive reduced access to both labor and mating markets, reinforcing perceptions of inequality across domains. Indeed, hypergamous mating dynamics—where women “marry up” to men of higher status than themselves—are widespread across cultures (Betzig, 1986, 1993; Dickemann, 1979; Nettle & Pollet, 2008; Van Leeuwen & Maas, 2010; Wooding et al., 2004). As a result, high- but not low-income men tend to experience the best mating prospects (Brooks, Blake, et al., 2022; Hopcroft, 2006, 2021). This pattern is reversed in women, who in hypergamous markets have fewer men of greater status than themselves to choose from (Brooks, Blake, et al., 2022). In simulation models these patterns are amplified by economic and gender equality (Brooks, Blake, et al., 2022), which worsen the mating success of low-income men and high-income women, respectively.

These points then suggest that labor markets and mating markets are inherently linked, which is supported by evidence from numerous studies. For example, higher female earnings have been found to decrease gains to marriage, hypothetically leading to reduced marriage rates and increased divorce rates (van der Klaauw 1996). Similarly, Blau et al. (2000) found that marriage rates were lower under better female labor market conditions and worse under better male labor market conditions. Meanwhile, Loughran (2002) found a positive relationship between men's wage inequality and women's age at first marriage in the United States between 1970 and 1990. More recently, Autor et al. (2019) examined the impact of large-scale labor demand shocks in the US, which decreased the relative earnings of men compared to women, finding that such negative shifts led to fewer marriages, reduced birth rates, and a greater share of single mothers.

While men's labor market access and women's economic dependence on men have important implications for both sexes’ successful access to the mating market, limited research examines individuals’ perceptions of such factors. With regard to labor market access, Taylor et al. (2012) examined ethnic and sex differences in perceived difficulty of acquiring a graduate job among final-year and recently graduated university students. Their results showed that male and female participants from all ethnic backgrounds perceived greater difficulty for women than men; however, female participants’ average ratings of job acquisition difficulty (for both sexes) were lower than male participants. Meanwhile, Price et al. (2014) investigated the relationship between perceptions of women's economic dependence and attitudes toward promiscuity, finding that male and female participants who perceived greater dependence were more opposed to short-term dating. They also found that women with higher incomes were less opposed to promiscuity, while men who reportedly out-earned their partner were more opposed. These findings align with the hypothesis of Gangestad & Simpson (2000) that women who are less dependent on male parental investment (such as provision of material resources) are more likely to be interested in short-term mating and value signals of genetic fitness (such as physical attractiveness) more highly.

In extreme cases, perceptions of inequities in access to the labor and mating markets can lead to harmful attitudes and behaviors both online and in the real world. This can be seen in online communities of “involuntarily celibate” men, commonly referred to as “incels,” who feel they have been excluded from the mating market due to their perceived unattractiveness and low earning capacity, criticizing women and society for their apparent preoccupation with status and good looks (Baele et al., 2021; Farrell et al., 2019). These perceptions can be harmful as they lead to extreme negative messaging online, which often includes justifying the use of violence toward women as a means to redistribute sexual resources (Baele et al., 2021; Blake et al., 2020). In a survey sample of incels collected by Blake et al. (2020), the majority of men had not completed any tertiary education and reported an annual income of less than AUD$60,000. Additionally, Brooks et al. (2022) found that incel-related tweets were more common in geographic areas where economic conditions worsened low status men's mating access. Such findings may indicate that these men are conscious of their relative socioeconomic status in their community and how this impacts their ability to compete and achieve success in the mating market. As these findings are limited to the extreme case of men who self-identify as incels, further research is needed to understand differences in perceptions of access to the labor and mating markets between sexes and across a broader sample.

Interestingly, growing scientific literature is now examining modern digital behaviors, like online dating, as channels for reproductive (fertility) access, allowing further insight into potential interplays arising from economic disparities. Online dating platforms uniquely enable researchers to study mating behaviors and preferences in a highly controlled yet dynamic environment, offering insights that are less accessible through traditional dating contexts. Unlike face-to-face interactions, online platforms provide extensive data on individual preferences, matching behaviors, and messaging patterns, allowing for a nuanced understanding of modern mating strategies, be they for extra-pair copulations, long term dyadic pair bonds, or even for reproduction both with and without a mate (i.e., gamete donation) (Whyte et al., 2017). Numerous studies have directly examined users’ profiles and messaging or matching behaviors to learn about their mating preferences and choices (Fiore et al., 2010; Hitsch et al., 2010; Lee et al., 2008; Skopek et al., 2011; Whyte & Torgler, 2017; Whyte, Chan, et al., 2018). Others have conducted surveys among online dating users to explore various aspects of human mating and sexuality (Chan et al., 2021; Whyte et al., 2019; Whyte et al., 2021). Given that online dating has recently become the predominant method human beings now find a partner (for Australia, see, e.g., Relationships Australia, 2023), this area of research is timely and pertinent.

This study is guided by hypotheses that perceptions of labor market access and women's economic dependence on men will vary significantly based on individual factors (e.g., sex, income, and education) and regional socio-economic conditions. Furthermore, we hypothesize that these perceptions will align with evolutionary theories of mate choice regarding reproductive costs, resource acquisition, and homogamous or hypergamous mating strategies, particularly in the context of economic inequality. This provides a framework for examining how labor and mating markets intersect in shaping individuals’ perceptions and behaviors.

Overall, this study provides new evidence on the cybermating-labor market relationship utilizing a large cross-sectional sample of the Australian public (*n *= 1072) currently engaged in the online dating market. We explore the influence of sex, other individual attributes, and market conditions on perceptions of men's ease of access to the labor market (opportunity), women's economic dependence on men (gender inequality), and relative reproductive opportunity (access) for both sexes.

## Data and Methods

### Data Collection

Research participants were recruited via email invitations sent to users of various Australian commercial dating websites operated by GIGA Pty Ltd (i.e., Adultmatchmaker.com.au, Aussiematchmaker.com, and theloveclub.com.au). This approach included contacting individuals who were currently and or previously active on these platforms but may have no longer been using them at the time of the survey (i.e., already found a mate), provided they had not unsubscribed from communications or deleted their accounts. Participants were required to be 18 years of age or older at the time of the survey to be included in the study. Participation was incentivized with two random prize draws of $500 AUD, as is commonly employed in behavioral economics research (Whyte et al., 2021). The research was conducted in accordance with the guidelines set forth by the Australian University Human Research Ethics Clearance (QUT Ethics Approval Number: 1600000221). Data collection for the survey was carried out between October 9, 2019 and December 23, 2019.

### Dependent Variables

Our study consisted of five key dependent variables relating to both economic and dating market access and opportunity. To capture perceptions of female economic dependence, participants were asked: “*In your community, how economically dependent are women on their male partners*?” on a scale from 0% (not dependent at all) to 100% (completely dependent). We assessed perceptions of men's labor market access by asking participants: “*In your community, how hard is it for men to ‘get a decent paying job/be economically successful’*?” on a scale from 0% (extremely difficult) to 100% (extremely easy). Perceptions of access to the reproductive market were captured by asking participants: “*In your community, how hard is it for men to find a date*?” and “*In your community, how hard is it for women to find a date*?” both ranked from 0% (extremely difficult) to 100% (extremely easy). To derive insights into perceived gender imbalances in reproductive market access, we computed the relative difference by subtracting the perceptions of women's dating accessibility from that of men (this variable will be referred to as “sex difference in perception on ability to find a date”). The term “find a date” was used for both questions as the simplest description of first steps for mating market access, without a need to differentiate between short- or long-term mating strategy objectives (i.e., copulation vs pair bonding and reproductive decision making). For the purposes of this study, differentiation between such strategies isn’t necessary in order to understand initial dating market “access” to reproductive opportunities.

### Quasi-independent Variables

Our analysis sought to understand the role of individual attributes and socioeconomic factors in shaping perceptions of access to the labor and mating markets. Individual-level characteristics included sex, age, education, and relative earning potential. At the market level, we examined income, unemployment, and sex ratio, in addition to geographical differences. Descriptive statistics for these variables are presented in Table A1.

**Figure 1.**
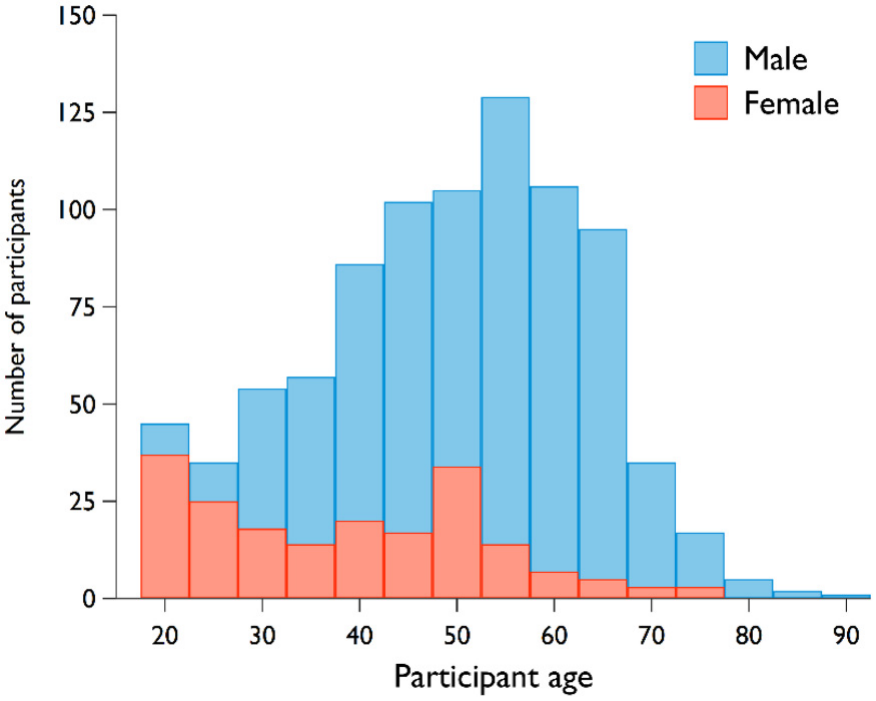
Age distribution by sex.

With regard to sex and age, the sample for this study included only those participants who described their sexual orientation as heterosexual (*N* = 1072) and identified their sex as either male (*N*_Male_ = 875) or female (*N*_Female_ = 197), aged between 18 and 81 years. The average age for male participants was 48.72 (SD = 14.03), with a median age range of 39 to 59 years. In contrast, female participants had an average age of 38.63 (SD = 14.57), with their median age ranging between 24 and 50 years. This demographic distribution aligns with established literature in online dating, which indicates a predisposition for older males to frequent online dating platforms more than their female counterparts. Furthermore, general sex ratios in online dating typically lean toward a male-dominated user base (Whyte, Chan et al., 2018). A detailed age distribution, categorized by sex, can be found in Figure 1.

Participants were asked to indicate their highest level of education, from “below Grade 10” up to “Doctor/PhD.. Some 85.11% of respondents had attained an education level of Year 12 or higher. Comparing the sexes, the proportion of females with a minimum of Year 12 education level was greater than that of males (91.44% versus 83.69%, respectively). Further, 5.35% of female respondents reported having completed doctoral studies, compared to 3.84% of males.

The survey also asked participants to self-rate their own earning potential, followed by their ideal long-term mate's earning potential (both on a scale from 1 to 10). To examine the role of relative earning potential in shaping perceptions of economic and mating access, we constructed the variable “Earning potential difference” (self—ideal long-term partner). In our sample, the mean earning potential difference among female participants was −0.57 (*N *= 145; SD* *= 2.31), indicating that on average, they rated their own earning potential lower than that of their ideal long-term partner. Conversely, male participants tended to rate their earning potential higher than that of their ideal long-term partner with a mean of 0.41 (*p *< .001).

We collected participants’ postcode of current residency to control for the influence of geographical and socioeconomic factors. Of the total number of participants in the sample, the majority were living in New South Wales (29.29%), closely followed by Queensland (27.24%) and Victoria (23.04%). Only five participants (0.47%) lived in the Northern Territory, with the remaining 19.96% living in South Australia, Western Australia, Tasmania, or the Australian Capital Territory (ACT). To capture differences between urban and regional areas, we created a dummy variable equal to 1 if living in a capital city area (i.e., developed (sub)urban areas as opposed to regional areas) and 0 otherwise. Across the states, 58.96% of participants reported living in a capital city at the time of the survey. Female participants were also more likely to reside in capital cities (69%), compared to male (57%, *p *= .002). This is in line with the Australian Bureau of Statistics data for 2021, with approximately 61% of the Australian population living in capital cities.

To evaluate the influence of local market conditions on perceptions of economic and mating access, and guided by relevant data availability, we integrated regional-level socioeconomic data from the Australian Bureau of Statistics (ABS) into our analysis (ABS, 2016, 2018–2019, 2019a, 2019b), which were linked to respondents and clustered by postcode utilizing geographic correspondences produced by ABS Geospatial Solutions (2016). One such variable was median personal income, reported in financial year 2018–2019 by Statistical Area Level 3 (SA3). We also took unemployment rate by Statistical Area Level 4 (SA4) from the 2016 census, and 2019 sex ratios by Statistical Area Level 2 (SA2) (males per 100 females). Compared to male participants, females in the sample on average lived in areas with higher median income (*M*_Female_* *= AU$56742; *M*_Male_ = AU$55241; *p *= .002) and slightly lower unemployment rate (*M*_Female_ = 5.4%; *M*_Male_* *= 5.7%; *p *= .067). There was no significant difference in average sex ratio between male and female participants (*M*_Female_* *= 98.56; *M*_Male_* *= 98.55; *p *= .972). We also constructed variables to capture the relative differences in income and unemployment rates between sexes by taking the logarithm of income differences (male-female) and rate differences for unemployment (male-female).

### Other Control Variables

Our study also controlled for participants’ relationship status, number of offspring, weekly sex frequency, difference between preferred age of ideal partner and own age, self-rated life satisfaction, attractiveness and health, and political views. Descriptive statistics for these variables are presented in Table A2.

We constructed a dummy variable equal to 1 if participants were in some form of relationship at the time of the survey, and 0 if they were single. Those who stated they were in a relationship included participants who reported being in a de facto relationship/cohabitating, engaged, or married, while the latter includes those who reported being divorced, separated, single, widowed, or other. In our sample, the majority of males (57.37%) and females (65.48%) were not in a relationship. When asked about offspring, 63.36% of respondents reported having at least one child. The mean number of children reported was 1.568, with males having a slightly higher mean than the females (*M*_Male_ = 1.650; *M*_Female_* *= 1.188, *p *= .001).

In terms of sexual frequency, we asked participants “In an average week, how many times would you have sex?”, on a scale ranging from zero to ten or more times. There was no significant difference in average weekly sex frequency between males (*M *= 1.87) and females (*M *= 2.00; *p *= .464). Participants indicated their ideal partner's age by selecting a single numeric value between 18 and 100. We constructed a variable labeled “Age of ideal partner difference” by subtracting one's own age from their preferred ideal partner's age. On average, male and female participants preferred partners younger than themselves; however, this difference was significantly greater among males (*M*_Female_* *= −0.49; *M*_Male_* *= −8.77; *p *< .001).

Since self-perception of attractiveness, health, and life satisfaction have been shown to influence mating preferences and behaviors in cyber settings (Whyte & Torgler, 2016; Whyte, Brooks, et al., 2018; Whyte, Chan, et al., 2018, 2019) participants were asked to self-assess their life satisfaction (happiness), overall health, and their perception of their own physical attractiveness using a scale ranging from 0 (*not attractive at all*) to 100 (*extremely attractive*), with incremental units of 1. On average, female participants rated themselves more highly than males in terms of life satisfaction (*M*_Female_* *= 72.35; *M*_Male_* *= 67.42; *p *= .010) and attractiveness (*M*_Female_* *= 65.03; *M*_Male_* *= 60.77; *p *= .003). Meanwhile, there was no significant difference regarding self-rated health (*M*_Female_* *= 70.93; *M*_Male_* *= 73.04; *p *= .154).

Finally, we included participants’ political views, as perceptions among conservative individuals for example may align with more traditional gender and societal norms. To capture political conservatism, we constructed four dummy variables (*Centralist*, *Left-wing**^2^***, *Non-partisan*, and *Other*), with *Right-wing**^3^*** as the reference group. The majority of participants in the sample aligned with right-wing, left-wing, or non-partisan political views; however, the distribution across these three groups varied between sexes. The proportion of individuals who aligned with right-wing views was greater among male participants (27.2%) compared to females (16%; *p *= .003). There was also a significant difference in the proportion of individuals who aligned with centralist views among males (8.9%) compared to females (2.6%; *p *= .007). Meanwhile, more females (41.7%) than males (27.4%) aligned with left-wing views (*p *< .001). There were no significant differences in the proportion of individuals who aligned with non-partisan (Male* *= 24.6%; Female* *= 30.1%; *p *= .150) or other political views (Male* *= 11.9%; Female* *= 9.6%; *p *= .423).

The survey also included all nine items from the 9-point response scale version of the Revised Sociosexuality Orientation Inventory (SOI-R) instrument (Penke & Asendorpf, 2008). Items were coded appropriately and aggregated to create a sum and an average score for each facet; that is, Behavior (items 1–3), Attitude (items 4–6), and Desire (items 7–9) as well as the total sum and average score (items 1–9). These SOI-R scores are not included in the primary analysis, but will be used to conduct additional robustness checks.

### Analysis

To explore sex differences in the perceptions of access to labor and reproductive markets, we first performed two-sample *t*-tests examining our five key dependent variables by sex. We then used ordinary least squares (OLS) regression to account for relevant socio-demographic and personal characteristics of our participants. The OLS analyses also control for regional level characteristics taken from the ABS, such as income, unemployment rates, and sex ratios.

## Results

### Descriptive Results

**Figure 2.**
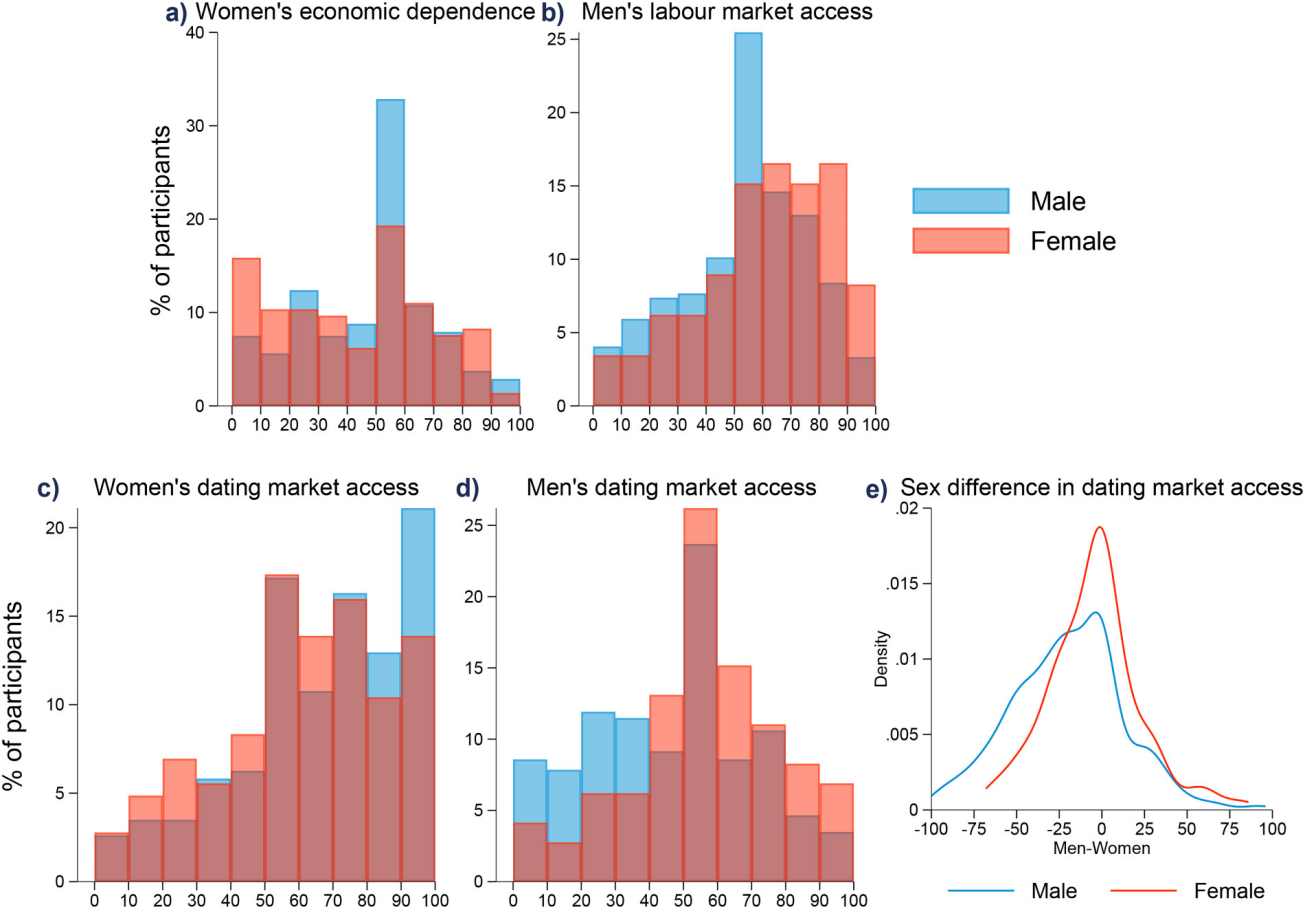
Distribution of perceptions on labor and dating market access, by sex. Bin size for (a)–(d) is 10. (e) The density estimates using the Gaussian kernel function with half-width of the kernel of 8.

Figure 2 illustrates how male and female participants perceive sex-specific access to the labor and dating markets across our five key variables of interest. Table 1 presents the results of our two-sample *t* tests and pairwise correlations for these variables.

**Table 1.** Descriptive Statistics of Perceptions on Labor and Reproductive Market Access.

Variables	Mean	SD	*N*	Correlation			
**Male participants**				(1)	(2)	(3)	(4)
(1) Women's economic dependence	45.64	22.44	694	1			
(2) Men's labor market access	52.04	22.31	691	0.095**	1		
(3) Women's dating market access	64.86	25.05	687	0.037	0.122***	1	
(4) Men's dating market access	44.91	23.88	688	0.142***	0.337***	0.089**	1
Dating market access (diff)	−19.95	33.05	687	0.074*	0.151***	−0.694***	0.656***
**Female participants**				(1)	(2)	(3)	(4)
(1) Women's economic dependence	41.22	26.32	145	1			
(2) Men's labor market access	59.52	24.03	145	0.017	1		
(3) Women's dating market access	59.55	24.81	144	0.128	0.325***	1	
(4) Men's dating market access	54.88	21.42	145	0.137*	0.268***	0.312***	1
Dating market access (diff)	−4.64	27.29	144	−0.010	−0.085	−0.664***	0.504***
**Sex differences**	Diff.	*t*-stat.	*p*-val.	*z*-stat.	*p*-val.		
Women's economic dependence	4.419	−2.09	.037	−1.677	.094		
Men's labor market access	−7.482	3.622	<.001	3.664	<.001		
Women's dating market access	5.307	−2.316	.021	4.523	<.001		
Men's dating market access	−9.967	4.647	<.001	−2.416	.016		
Dating market access (diff)	−15.32	5.2	<.001	5.34	<.001		

*Note.* ***p < .01, **p < .05, *p < .1. *t* and *z* are the test statistics of the two-sample *t*-test and the Wilcoxon rank-sum (Mann–Whitney) test, respectively.

Our analysis reveals notable differences in perceptions between the sexes. Relating to economic access, female participants perceived women as less economically dependent on men (*M *= 41.2) than male participants (*M *= 45.6) (Figure 2, panel a, *p *= .037). At the same time, female participants perceived males as having greater access to the labor market (*M *= 59.5) compared to males (*M *= 52) (panel b, *p *< .001). Compared to female participants, male participants were more likely to believe that women had greater access to the dating market than men did (panel c, Diff* *= 5.3, *p = *.021 and panel d, Diff = −9.97, *p *< .001, see Table 1). When comparing male and female participants’ difference in perceptions of dating market access (male–female), both sexes thought that females found it relatively easier to find a date (panel e, *M*_m_* *= −19.95, *M*_f_ = −4.64). Male participants perceived that males had less access to the reproductive market than did females, indicating a larger gap in reproductive market access perceptions between the sexes (Diff = −15.3, *p *< .001).

Our study also found (see Table 1) sex differences in the form of correlations among the four perceptions. Most pairwise correlations were positive for both male and female participants. Perceptions of women's economic dependence and dating market access were not correlated for either sex. Correlations between perceptions of females’ dating market access and males’ labor (*ρ*_f_* *= 0.325 vs *ρ*_m_* *= 0.122) or dating (*ρ*_f_ = 0.312 vs *ρ*_m_ = 0.089) market access were larger among female participants.

### Regressions Results

#### Perceptions on Economic Access

Our regression analysis investigates the factors influencing perceptions of sex-specific labor market access, beginning with perceptions of women's economic dependence on men (Table 2), and perceptions of the ease of finding a job for men (Table 3). The first model specification only considers the sex of the participant, with no other quasi-independent variables included. In specification 2, we add further individual attributes (age, education, and earning potential difference), and the capital city dummy as a proxy to capture differences between those living in urban versus regional areas, as well as additional controls (relationship status, number of children, weekly frequency of sex, age of ideal partner difference, self-rated life satisfaction, attractiveness, and health, and political views), standardized within sex. In the third specification, the capital city dummy is substituted for socioeconomic factors (log median income, unemployment rate, log difference in income, difference in unemployment rate, and sex ratio). The fourth and fifth specifications include the same variables as the third specification, isolated by male participants (4) and female participants (5), allowing for more specific analyses within each sex category.

**Table 2.** Perception of Women's Economic Dependence on Men.

Dependent variable:	All			Males	Females
Women's economic dependence	(1)	(2)	(3)	(4)	(5)
**Quasi-independent variables (individual)**					
Male	4.42*	4.26*	3.78		
	(1.91)	(1.84)	(1.62)		
Age		2.09*	1.87*	0.08	0.32
		(1.95)	(1.70)	(1.02)	(1.09)
Education		−2.22***	−2.06**	−1.84***	1.00
		(−2.67)	(−2.48)	(−3.56)	(0.60)
Earning potential difference		−1.46*	−1.47*	−0.25	−2.59***
		(−1.74)	(−1.73)	(−0.63)	(−3.16)
**Quasi-independent variables (market-level)**					
Capital city		−2.36			
		(−1.41)			
ln(Income)			−14.64*	−1.92	−82.92***
			(−1.79)	(−0.22)	(−3.31)
Unemployment rate			0.02	−0.39	1.49
			(0.04)	(−0.72)	(0.94)
Log difference in income (M-F)			20.43**	22.31**	−11.56
			(2.08)	(2.08)	(−0.38)
Difference in unemployment rate (M-F)			0.19	0.13	1.02
			(0.50)	(0.32)	(0.88)
Sex ratio (M/F)			−0.13	−0.06	−0.58
			(−0.96)	(−0.45)	(−1.54)
**Other control variables**					
In a relationship		−0.41	−0.45	−0.82	−8.39*
		(−0.50)	(−0.54)	(−0.45)	(−1.74)
# Children		−0.39	−0.46	−0.10	−2.22
		(−0.43)	(−0.51)	(−0.16)	(−0.98)
Frequency of sex (weekly)		−0.22	−0.24	−0.22	0.67
		(−0.24)	(−0.27)	(−0.54)	(0.68)
Age of ideal partner difference		2.50***	2.53***	0.23*	0.92**
		(2.76)	(2.77)	(1.95)	(2.26)
Self-rated life satisfaction		−0.52	−0.65	−0.03	0.10
		(−0.50)	(−0.61)	(−0.62)	(0.82)
Self-rated attractiveness		0.45	0.64	0.02	0.04
		(0.43)	(0.62)	(0.27)	(0.32)
Self-rated health		−0.42	−0.53	−0.06	0.14
		(−0.42)	(−0.53)	(−1.01)	(1.15)
Centralist political views		−1.13	−1.01	−2.56	−10.00
		(−1.12)	(−0.97)	(−0.68)	(−0.56)
Left-wing political views		−3.38***	−3.26***	−6.90***	−10.40
		(−3.60)	(−3.42)	(−3.05)	(−1.55)
Non-partisan political views		−2.76***	−2.72***	−5.63**	−10.84
		(−2.72)	(−2.66)	(−2.21)	(−1.59)
Other political views		−1.81**	−2.09**	−6.82**	−7.20
		(−1.97)	(−2.29)	(−2.31)	(−0.81)
Constant	41.22***	44.84***	210.68**	89.26	980.21***
	(19.47)	(16.76)	(2.32)	(0.91)	(3.52)
State FE	No	Yes	Yes	Yes	Yes
*N*	839	838	820	680	140
Prob.* > F*	0.056	0.000	0.000	0.000	0.000
*R^2^*	0.005	0.064	0.071	0.079	0.263
*R^2^* adj.	0.004	0.037	0.039	0.043	0.101

*Note.* OLS regressions. Standard errors (in parentheses) are clustered at the postcode level.

*p < .1; **p < .05; ***p < .01.

**Table 3.** Perception of Men's Ability to Get a Decent Paying Job and be Economically Successful.

Dependent variable:	All			Males	Females
men's labor market access	(1)	(2)	(3)	(4)	(5)
**Quasi-independent variables (individual)**					
Male	−7.48***	−7.57***	−7.47***		
	(−3.41)	(−3.42)	(−3.29)		
Age		−1.10	−1.30	−0.08	−0.20
		(−0.95)	(−1.11)	(−0.86)	(−0.69)
Education		0.52	0.37	0.25	1.08
		(0.63)	(0.44)	(0.45)	(0.65)
Earning potential difference		0.55	0.49	0.32	−1.07
		(0.60)	(0.53)	(0.74)	(−1.15)
**Quasi-independent variables (market-level)**					
Capital city		2.79			
		(1.64)			
ln(Income)			21.79***	23.23***	−3.22
			(2.64)	(2.69)	(−0.10)
Unemployment rate			0.36	0.48	0.66
			(0.87)	(1.15)	(0.43)
Log difference in income (M-F)			−5.46	2.08	−45.92
			(−0.57)	(0.20)	(−1.46)
Difference in unemployment rate (M-F)			0.50*	0.30	2.35*
			(1.73)	(0.98)	(1.92)
Sex ratio (M/F)			0.07	0.04	0.02
			(0.54)	(0.33)	(0.04)
**Other control variables**					
In a relationship		1.07	1.01	2.89	−4.07
		(1.34)	(1.24)	(1.63)	(−0.98)
# Children		1.55	1.80	1.32*	−0.65
		(1.45)	(1.65)	(1.80)	(−0.36)
Frequency of sex (weekly)		0.95	0.87	0.35	1.11
		(0.99)	(0.89)	(0.79)	(1.01)
Age of ideal partner difference		0.45	0.21	0.08	−0.52
		(0.45)	(0.21)	(0.61)	(−1.23)
Self-rated life satisfaction		1.97*	2.00*	0.09**	0.06
		(1.90)	(1.90)	(2.01)	(0.44)
Self-rated attractiveness		0.83	0.75	0.01	0.12
		(0.81)	(0.71)	(0.10)	(0.77)
Self-rated health		−0.23	−0.13	0.02	−0.13
		(−0.23)	(−0.12)	(0.43)	(−0.92)
Centralist political views		−1.23	−1.37	−7.86**	22.90***
		(−1.38)	(−1.48)	(−2.31)	(2.63)
Left-wing political views		−2.70***	−2.66***	−6.66***	1.16
		(−2.97)	(−2.87)	(−2.99)	(0.19)
Non-partisan political views		−2.91***	−2.71***	−7.53***	3.85
		(−2.94)	(−2.71)	(−3.06)	(0.55)
Other political views		−1.27	−1.13	−3.89	3.13
		(−1.49)	(−1.31)	(−1.34)	(0.37)
Constant	59.52***	59.33***	−185.06**	−212.80**	101.10
	(30.02)	(22.38)	(−2.06)	(−2.27)	(0.30)
State FE	No	Yes	Yes	Yes	Yes
*N*	836	835	817	677	140
Prob.* > F*	0.001	0.000	0.000	0.000	0.002
*R^2^*	0.015	0.073	0.082	0.096	0.146
*R^2^* adj.	0.014	0.047	0.051	0.060	−0.041

*Note.* OLS regressions. Standard errors (in parentheses) are clustered at the postcode level.

*p < .1; **p < .05; ***p < .01.

Specifications 1 and 2 (Table 2) replicate the result that male participants perceive women to be more economically dependent on men than female participants. However, this effect becomes statistically insignificant (*p *= .105) when we substitute the capital city dummy for macro-economic variables in specification 3. In Table 3, we see consistent statistically significant sex differences in perceptions of men's labor market access across specifications 1 to 3. Specifically, we find male participants’ perceptions of their ease of labor market access to be around 7.5% lower, on average, compared to female participants (*p *= .001).

Interestingly, our regression analysis shows more highly educated male online daters are more likely to believe women are less economically dependent on their male partners (*p *< .001, Table 2, specification 4). Further, female participants who express a preference for a partner with a higher earning potential than their own tend to state a higher perception of women being more economically dependent on their male partners (*p *= .002, Table 2, specification 5). However, there are no statistically significant effects for age, education, or earning potential difference with regard to perceptions of men's ability to get a decent paying job (Table 3, specifications 2–5).

In relation to market-level factors, our analysis reveals that male participants living in areas with a higher sex difference in income tend to perceive women as more economically dependent on men (*p *= .038, Table 2, specification 4). In contrast, female participants living in higher income areas state lower perceptions of women's economic dependence (*p *= .001, Table 2, specification 5). Male participants living in higher-income areas are more likely to believe that men have easier access to job opportunities (*p *= .007, Table 3, specification 4). Meanwhile, female participants living in areas with higher sex differential unemployment rate are more likely to perceive higher labor market access for men (*p *= .057, Table 3, specification 5).

Among the additional controls, the difference between the age of participants’ ideal partner and their own was found to have a significant positive effect on perceptions of women's economic dependence among both male (*p *= .052, Table 2, specification 4) and female participants, though to a greater extent among the latter (*p *= .026, Table 2, specification 5). Interestingly, male participants who aligned with left-wing (*p *= .002), non-partisan (*p *= .028), or other political views (*p *= .021), perceived lower economic dependence of women than right-wing males (Table 2, specification 4). Meanwhile, centralist (*p *= .022), left-wing (*p *= .003), and non-partisan males (*p *= .002) stated lower perceptions of men's labor market access (Table 3, specification 4). Conversely, female participants who aligned with centralist views perceived greater ease of access to the labor market for men, compared to right-wing females (*p *= .010; Table 3, specification 5). Male participants’ perceptions on men's job market access were also positively correlated with the number of offspring (*p *= .073) and self-rated life satisfaction (*p *= .045) (Table 3, specification 4).

Finally, in both Tables 2 and 3, our sex differentiated R-squared results show these models account for much more of the variance in women's perceptions compared to men's (Table 2, *R*^2^, *M *= 0.079 and *F *= 0.263; Table 3, *R*^2^, *M *= 0.096 and *F *= 0.146).

#### Perceptions on Dating Access

In Tables 4 and 5, we explore participants’ perceptions of female's and male's ability to find a date. In both tables we see a statistically significant sex difference in that, on average, male participants (when compared to females) believe it is harder for men (*p *< .001) but easier for women (*p *< .023) to find a date (specifications 1 to 3).

**Table 4.** Perception of Women's Ability to Find a Date.

Dependent variable:	All			Males	Females
women's dating market access	(1)	(2)	(3)	(4)	(5)
**Quasi-independent variables (individual)**					
Male	5.31**	5.30**	5.64**		
	(2.27)	(2.20)	(2.28)		
Age		−3.35***	−3.31***	−0.26***	−0.00
		(−2.80)	(−2.73)	(−2.81)	(−0.01)
Education		0.14	0.04	−0.05	−0.02
		(0.15)	(0.05)	(−0.08)	(−0.01)
Earning potential difference		1.58	1.72*	0.78*	0.37
		(1.64)	(1.74)	(1.78)	(0.39)
**Quasi-independent variables (market-level)**					
Capital city		−0.08			
		(−0.04)			
ln(Income)			15.27*	10.85	63.41**
			(1.69)	(1.10)	(2.14)
Unemployment rate			−0.10	−0.53	3.39***
			(−0.22)	(−1.02)	(2.77)
Log difference in income (M-F)			8.11	17.72	−67.63**
			(0.73)	(1.48)	(−2.02)
Difference in unemployment rate (M-F)			−0.09	−0.18	0.64
			(−0.26)	(−0.48)	(0.49)
Sex ratio (M/F)			0.06	0.13	−0.47
			(0.39)	(0.80)	(−0.91)
**Other control variables**					
In a relationship		−0.08	−0.11	−0.19	−1.97
		(−0.09)	(−0.12)	(−0.10)	(−0.44)
# Children		0.16	0.30	0.37	−1.35
		(0.14)	(0.26)	(0.48)	(−0.61)
Frequency of sex (weekly)		0.65	0.56	0.07	0.73
		(0.64)	(0.53)	(0.14)	(0.67)
Age of ideal partner difference		−1.23	−1.46	−0.23*	0.20
		(−1.20)	(−1.40)	(−1.65)	(0.46)
Self-rated life satisfaction		−0.57	−0.38	−0.02	0.08
		(−0.53)	(−0.35)	(−0.45)	(0.58)
Self-rated attractiveness		−1.59	−1.79	−0.11*	−0.12
		(−1.45)	(−1.59)	(−1.72)	(−0.78)
Self-rated health		1.97*	1.91*	0.11*	0.10
		(1.85)	(1.77)	(1.77)	(0.63)
Centralist political views		1.23	1.39	4.70	10.09
		(1.28)	(1.39)	(1.17)	(0.62)
Left-wing political views		0.69	0.72	0.64	11.52*
		(0.65)	(0.67)	(0.24)	(1.81)
Non-partisan political views		−0.37	−0.23	−2.22	12.75*
		(−0.34)	(−0.21)	(−0.83)	(1.70)
Other political views		−0.05	0.06	−2.22	18.30*
		(−0.05)	(0.05)	(−0.64)	(1.80)
Constant	59.55***	58.65***	−116.76	−59.93	−600.97*
	(28.53)	(20.53)	(−1.19)	(−0.56)	(−1.84)
State FE	No	Yes	Yes	Yes	Yes
*N*	831	830	812	673	139
Prob.* > F*	0.024	0.043	0.006	0.005	0.000
*R^2^*	0.006	0.043	0.050	0.052	0.192
*R^2^* adj.	0.005	0.016	0.017	0.014	0.013

*Note.* OLS regressions. Standard errors (in parentheses) are clustered at the postcode level.

*p < .1; **p < .05; ***p < .01.

**Table 5.** Perception of Men's Ability to Find a Date.

Dependent variable:	All			Males	Females
men's dating market access	(1)	(2)	(3)	(4)	(5)
**Quasi-independent variables (individual)**					
Male	−9.97***	−9.95***	−9.77***		
	(−5.23)	(−5.07)	(−4.86)		
Age		−0.28	−0.44	0.04	−0.31
		(−0.25)	(−0.38)	(0.42)	(−1.28)
Education		−1.80**	−1.61*	−0.73	−2.19*
		(−2.03)	(−1.77)	(−1.18)	(−1.74)
Earning potential difference		1.82**	1.87**	0.82**	−0.25
		(2.12)	(2.13)	(2.01)	(−0.39)
**Quasi-independent variables (market-level)**					
Capital city		6.47***			
		(3.53)			
ln(Income)			23.20***	24.07**	11.85
			(2.62)	(2.46)	(0.55)
Unemployment rate			0.54	0.25	2.24**
			(1.15)	(0.49)	(2.18)
Log difference in income (M-F)			−9.91	−11.68	−7.15
			(−0.90)	(−0.94)	(−0.27)
Difference in unemployment rate (M-F)			−0.28	−0.46	0.82
			(−0.83)	(−1.30)	(0.99)
Sex ratio (M/F)			−0.12	−0.09	−0.38
			(−0.79)	(−0.53)	(−1.40)
**Other control variables**					
In a relationship		−0.00	−0.36	−1.46	1.43
		(−0.00)	(−0.42)	(−0.79)	(0.35)
# Children		2.21*	2.07*	1.79**	−3.39**
		(1.93)	(1.79)	(2.28)	(−2.44)
Frequency of sex (weekly)		1.67*	1.43	0.86*	−0.31
		(1.81)	(1.50)	(1.83)	(−0.44)
Age of ideal partner difference		0.41	0.37	0.14	−0.61
		(0.41)	(0.37)	(1.00)	(−1.55)
Self-rated life satisfaction		0.26	0.14	−0.00	−0.04
		(0.25)	(0.14)	(−0.03)	(−0.38)
Self-rated attractiveness		1.17	1.57	0.09	0.06
		(1.10)	(1.47)	(1.29)	(0.61)
Self-rated health		1.41	1.16	0.09	0.01
		(1.33)	(1.08)	(1.53)	(0.06)
Centralist political views		1.07	1.42	2.78	34.07***
		(1.17)	(1.49)	(0.72)	(3.60)
Left-wing political views		0.43	0.52	−1.53	18.69***
		(0.43)	(0.53)	(−0.65)	(3.03)
Non-partisan political views		−1.06	−1.14	−5.72**	19.20***
		(−1.02)	(−1.07)	(−2.15)	(2.89)
Other political views		0.78	0.45	1.21	13.26
		(0.87)	(0.50)	(0.40)	(1.57)
Constant	54.88***	53.34***	−185.48*	−218.58**	−30.04
	(33.60)	(22.02)	(−1.90)	(−2.05)	(−0.13)
State FE	No	Yes	Yes	Yes	Yes
*N*	833	832	814	674	140
Prob.* > F*	0.000	0.000	0.000	0.000	0.000
*R^2^*	0.025	0.087	0.085	0.088	0.323
*R^2^* adj*.*	0.024	0.061	0.054	0.052	0.174

*Note.* OLS regressions. Standard errors (in parentheses) are clustered at the postcode level.

*p < .1; **p < .05; ***p < .01.

Similar to economic access, we also find biological, socio-demographic and economic characteristics shape perceptions of dating market access. For example, when analyzing the two samples separately (specifications 4 and 5), we see that older male participants believe women have lower dating market (*p *= .005) access while a linear age effect is not visible for females (*p *= .992). And male participants who rate their own earning potential higher than their ideal partner have more positive evaluations concerning the ease of finding a date for men (*p *= .045).

We find that while males’ perceptions of women's dating market access are not shaped by economic characteristics, female participants who live in areas with higher median income (*p *= .034), and with lower economic gender disparity in income (*p *= .046) are more likely to believe that women have higher dating market access. Meanwhile, median income (*p *= .014) and local unemployment rate (*p *= .032) are positively correlated with males’ and females’ perception of men's reproductive access, respectively. Additionally, when observing the total sample, participants living in capital cities are found to have higher perceptions of men's labor market access (*p *< .001, Table 5, specification 2).

Among the control variables, age difference between ideal partner and self (*p *= .099) and self-rated attractiveness (*p *= .087) have a slight negative impact on male participants’ perceptions of women's ability to find a date (Table 4, specification 4). Meanwhile, the opposite effect is seen for self-rated health (*p *= .077). While males’ political views do not have significant effects, females who express left-wing (*p *= .073), non-partisan (*p *= .091), or other political views (*p *= .074) have more positive perceptions of women's dating market access, compared to right-wing females (Table 4, specification 5).

The correlation between number of children and the perception of men's dating market access is positive for male participants (*p *= .023) but negative for females (*p *= .016). Similarly, male participants with non-partisan political views perceive greater difficulty for men in finding a date compared to right-wing males (*p *= .032, Table 4, specification 4), whereas the opposite effect is observed for females (*p *= .005, Table 4, specification 5). Female participants who align with centralist (*p *< .001) or left-wing political views (*p *= .003) also state higher perceptions of men's dating market access compared to right-wing females. Additionally, males’ perceptions of men's dating market access are positively correlated with their self-reported weekly sexual activity (*p *= .068).

Furthermore, when exploring differences in the relative perception of dating market access (men–women; Table 6), we find that male participants believe that men have relatively more difficulty than women in finding a date, compared to female participants (Table 6, *p *< .001). However, this effect is reduced for participants living in capital cities (*p *= .007, specification 2), and for older male participants (*p *= .012, specification 4).

**Table 6.** Sex Difference in the Perception of Ability to Find a Date.

Dependent variable:	All			Males	Females
gender difference in dating market access	(1)	(2)	(3)	(4)	(5)
**Quasi-independent variables (individual)**					
Male	−15.32***	−15.33***	−15.51***		
	(−5.62)	(−5.37)	(−5.23)		
Age		3.05**	2.85*	0.29**	−0.31
		(2.01)	(1.87)	(2.53)	(−0.92)
Education		−1.93	−1.64	−0.67	−2.17
		(−1.62)	(−1.36)	(−0.81)	(−1.14)
Earning potential difference		0.24	0.16	0.04	−0.59
		(0.20)	(0.13)	(0.08)	(−0.62)
**Quasi-independent variables (market-level)**					
Capital city		6.57***			
		(2.70)			
ln(Income)			8.26	13.22	−48.90
			(0.71)	(1.04)	(−1.58)
Unemployment rate			0.64	0.78	−1.11
			(1.07)	(1.15)	(−0.73)
Log difference in income (M-F)			−18.51	−29.42*	54.77
			(−1.32)	(−1.84)	(1.39)
Difference in unemployment rate (M-F)			−0.19	−0.28	0.07
			(−0.43)	(−0.56)	(0.05)
Sex ratio (M/F)			−0.18	−0.22	0.07
			(−0.89)	(−1.03)	(0.12)
**Other control variables**					
In a relationship		0.06	−0.26	−1.27	3.09
		(0.06)	(−0.23)	(−0.50)	(0.59)
# Children		2.06	1.78	1.42	−2.02
		(1.57)	(1.36)	(1.51)	(−0.94)
Frequency of sex (weekly)		1.00	0.85	0.79	−1.09
		(0.76)	(0.64)	(1.23)	(−1.04)
Age of ideal partner difference		1.63	1.82	0.37**	−0.81*
		(1.39)	(1.52)	(2.30)	(−1.80)
Self-rated life satisfaction		0.84	0.55	0.02	−0.10
		(0.63)	(0.40)	(0.30)	(−0.72)
Self-rated attractiveness		2.76*	3.37**	0.20**	0.17
		(1.96)	(2.35)	(2.16)	(1.16)
Self-rated health		−0.55	−0.74	−0.02	−0.08
		(−0.39)	(−0.51)	(−0.21)	(−0.50)
Centralist political views		−0.16	0.03	−1.91	23.65*
		(−0.13)	(0.03)	(−0.36)	(1.89)
Left-wing political views		−0.24	−0.18	−2.17	7.55
		(−0.17)	(−0.13)	(−0.63)	(0.95)
Non-partisan political views		−0.69	−0.91	−3.50	6.47
		(−0.47)	(−0.61)	(−0.95)	(0.66)
Other political views		0.83	0.40	3.44	−4.87
		(0.66)	(0.31)	(0.81)	(−0.42)
Constant	−4.64**	−5.21	−72.14	−158.71	544.01
	(−2.02)	(−1.43)	(−0.56)	(−1.12)	(1.62)
State FE	No	Yes	Yes	Yes	Yes
*N*	831	830	812	673	139
Prob.* > F*	0.000	0.000	0.000	0.000	0.059
*R^2^*	0.032	0.082	0.081	0.074	0.188
*R^2^* adj.	0.030	0.056	0.050	0.037	0.009

*Note.* OLS regressions. Standard errors (in parentheses) are clustered at the postcode level.

*p < .1; **p < .05; ***p < .01.

With regard to age difference between ideal partner and self, a positive effect is seen among male participants (*p *= .022, specification 4), while a negative effect is found for female participants (*p *= .075, specification 5). Additionally, male participants with higher self-rated attractiveness (*p *= .031) perceive the sex difference in dating access to be less negative than other males. Meanwhile, female participants who align with centralist political views perceive a greater sex difference compared to right-wing females (*p *= .061).

#### Non-Linearity in Age

**Figure 3.**
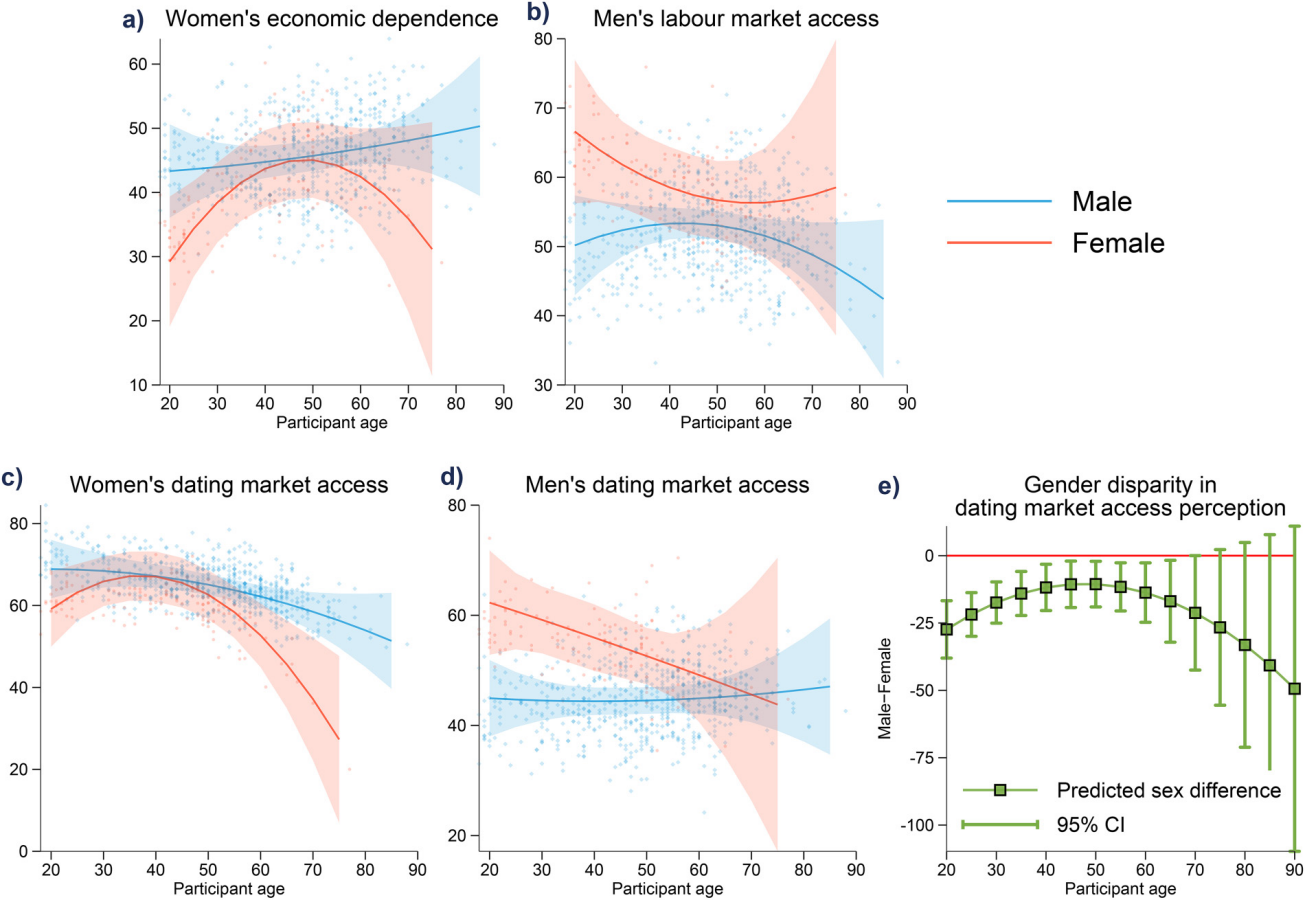
Perceptions on labor and dating market access across age, by sex. Solid lines in (a)–(d) represent the average predicted outcome based on participants’ age and sex. Shaded areas represent the 95% prediction intervals. (e) The estimated sex difference in gender gap of dating access perception.

Our rich data set allows us to provide a more nuanced analysis of how perceptions differ across age for both sexes. To do so, we estimate a set of regression models that include interaction terms between participants’ sex and age (Tables A3 and A4). To capture potential non-linearity in the age effects, we included age squared as interaction terms. We visualize the regression results in Figure 3 by showing the predicted levels of perceptions on labor (panels a and b) and dating market access (panels c and d) across age and sex, controlling for other factors. In panel e, we show how the sex difference in the perception of relative abilities of finding a date (between men and women) change across age.

In general, we find that male participants’ perceptions of economic and reproductive access are not age dependent. Meanwhile, older female participants state higher perceptions of women's economic dependence. Younger female participants (18 years to late 30s) believe it is easier for the women in their community to find a date, while women beyond that age group believe it is increasingly harder. Female participants’ perceptions of men's economic and reproductive access do not appear highly dependent on age, although the difference in perceptions of men's and women's dating market access does appear larger for the younger cohort, but this is slightly offset as they get older.

#### Robustness Check

We recognize that participants’ perceptions of dating market access may vary depending on whether they are searching for a short-term or long-term mating partner. To assess this, we conducted a robustness check (Table A5) using SOI-R scores as a proxy, where lower scores typically indicate more investment in monogamous long-term relationships, while individuals with higher scores tend toward more unrestricted, short-term mating strategies (Gangestad & Simpson, 2000; Penke & Asendorpf, 2008; Schmitt, 2005). The variable used was the individual's average score across all nine SOI-R items.

SOI-R scores are found to have a significant positive effect on perceptions of women's ability to find a date among both male and female participants (Table A5, specifications 1 and 2), which suggests that individuals who are more open to short-term mating believe it is easier for women to find a date, compared to those with more restricted sociosexual orientations. One explanation may be that individuals’ social communities are largely made up of women whose sociosexual tendencies are similar to their own, and hence their perceptions are anchored to that reference group. However, no such relationship is found for perceptions of men's ability to find a date (specification 3 and 4). Effects are no longer seen for the variables “Earning potential difference” and “Age of ideal partner difference” in specification 1 as well as log income in specification 2, as these were only significant at the 10% level in the original model.

## Discussion

Inequality in the labor market can have significant effects for men and women's perceptions, preferences, behaviors, and motivations (Becker & Lewis, 1973; Brooks, Blake, et al., 2022). Socio-economic and labor market differences also play a significant role in shaping the way that gender roles are perceived and culturally enforced, and how people with different resource endowments and opportunities identify with such (Whyte, Brooks, et al., 2018; Whyte, Chan, et al., 2018).

Across our five key variables, we identify significant labor market differences linked to perceptions of mating market access. For example, women in higher-income areas perceive less economic dependence on men, while men in areas with higher income levels report greater economic success and dating ease. These findings logically stem from both populations’ greater financial endowment, relative to the variable of interest. These results are further qualified in our aspirational self-report “earning difference” variable (expected earning potential difference between themselves and their ideal long-term partner), being that males who expect to earn the same or more than their ideal female mating partner believe it is easier for men to find a date. In turn, females who expect to earn the same or more than their ideal male partner believe women are less economically dependent on their male partners. Such findings conveniently punctuate the role and importance of financial resources (for both sexes) when engaging in the reproductive marketplace.

We also find that higher unemployment rates show a positive relationship with females’ perception of women's and men's ability to find a date, and that living in capital cities (for both sexes) translates to increases in perceptions of men's ability to find a date. Analogous to the labor market, where high unemployment rates indicate an oversupply of labor and low rates suggest a shortage, a logical inference is discernible in the mating market. Specifically, during periods of high unemployment, the opportunity cost may naturally elevate the mating market supply for both males and females. These may be two-fold, by either having greater disposable time to search and meet potential mates, or an oversupply that forces market participants to lower their own expected value and/or their market expectation for a suitable mate.

Our study also provides novel findings relating to more accentuated differences in female's perceptions (relative to male's) at different life stages for both female's economic dependence on men and female's ability to find a date. Firstly, regarding women's economic dependence, both males and females in the middle age bracket (35–55 years) share similar perspectives. However, this concave polynomial shape (Figure 3a) shows that both younger and older women believe that women are significantly less economically dependent on men than men do. Secondly, relating to women's ability to find a date, we again see a concave polynomial shape (Figure 3c) demonstrating younger women's (18–35 years) increasing belief in ease of dating access followed by a significant and sharp decline from 40 to 75+ years. That is, women appear to state greatest economic dependence on males across the years of childbirth and child rearing (i.e., 30–40s in developed economies). Additionally, female's perception of their reproductive (dating) access increases across the years of peak fertility (18–35 years), then sharply declines. Both findings demonstrate greater plasticity in perceptions of female market access at different life stages. These findings align with evolutionary perspectives on resource acquisition and mating preferences, highlighting how perceptions of labor and mating market access vary by sex, life stage, and socio-economic conditions. For instance, hypergamous mating dynamics—where women tend to seek partners with higher resource endowments—can explain why perceptions of economic dependence and reproductive opportunities differ by sex and life stage. As women achieve greater economic independence, their reliance on long-term partnerships for resource acquisition may decrease, potentially shifting mating preferences and strategies. Similarly, men's perceptions of dating market access may reflect competition for resources and status, which are critical factors in evolutionary mating strategies. While our findings emphasize perceptions of labor and mating market dynamics, future research could explore the extent to which these perceptions align with objective measures, such as income levels, employment rates, or financial dependence during key life stages. For instance, women's perception of greater economic dependence on men during the years of child rearing may reflect not only their own expectations, but also broader societal and cultural norms regarding resource provision. These perceptions may, in turn, influence behaviors and decision-making, such as preferences for long-term partnerships or career investments. Understanding the potential feedback loop between perceptions and actual economic or social conditions could provide richer insights into these dynamics.

The current study is not without limitations. Firstly, self-reported measures in sex, gender, and sexuality research can be problematic as the sensitivity of the questions and responses are impacted by individuals’ own preferences, biology, behavior, and related biases (Chan et al., 2021; Wiederman, 2001). For example, lifetime sexual partner numbers are subject to reporting biases influenced by cultural norms, societal expectations, and individual motivations, particularly in the context of the sexual double standard, may introduce variability that could affect the generalizability of our findings. Another limitation pertains to the overrepresentation of men in our sample, which reflects broader trends observed in online dating platforms, including those geared toward long-term relationships. This gender imbalance may stem from men's greater willingness to engage with digital dating solutions or perceived disadvantages in traditional dating markets. While this overrepresentation aligns with the general population using these platforms, it limits the generalizability of our findings, particularly regarding perceptions of access to mating markets by the general population not involved in online dating. An additional limitation relates to the interpretation of “dating access.” The survey question, “In your community, how hard is it for [men/women] to find a date?” was intentionally broad to capture general perceptions of dating ease. However, this generality may have introduced variability in responses due to individual preferences, biases, or gendered expectations. For instance, men and women may have interpreted the question differently based on their mating strategies (e.g., preferences for short-term vs long-term relationships) or societal norms. While this broad framing allowed for wide applicability, it also creates some ambiguity in understanding the specific contexts participants considered, which warrants further investigation in future research. Our reliance on self-reported perceptions introduces inherent biases, as these may not always align with objective realities. For example, perceptions of economic dependence and dating access may reflect societal norms or individual biases rather than actual conditions. Future research should validate these perceptions against objective measures, such as income or employment data, to provide a more comprehensive understanding. Finally, as cyber mate choices behavior and labor market conditions are dynamic, it is appropriate to note that the data collected for analysis in this study were prior to the COVID19 pandemic. Future cyber mate choice research would do well to incorporate and even quantify any impacts the global pandemic has had on cyber-mating behavior.

Mate selection presents a dual-faceted matching challenge, involving not only identifying a desired partner but also being desirable to them. This search complexity is further amplified in contemporary society where inequities in access and economic (dis)advantage can intensify the costs and restrictions of one's pursuit. Such complications can be problematic as the anger or frustration stemming from perceived or actual inequalities in access to mating markets can unleash substantial negative externalities on both the individuals and society at large (Blake et al., 2020; Blake & Brooks, 2019). Our study discerns variances in socio-economic factors, such as education level and parenthood, in relation to perceptions of economic and dating market access among Australian online daters of both sexes. Moreover, pivotal resource endowment markers, like income and unemployment, reveal perceptual disparities in both access and equity for both sexes. However, our findings underscore that perceptions of access to economic and mating markets exhibit notably more variation based on biological age (particularly for women) than any other variable. The discernible variances conceive avenues for future research, inviting scholars to delve deeper into understanding the underpinnings of such disparities and their ramifications on mating strategies and societal structures.

In general, integrating insights from economics, (evolutionary) psychology, and sociology can help to forge a comprehensive understanding of how disparities in perceived access to economic and mating markets sculpt individual perceptions, behaviors, and societal norms. Future research should investigate the reciprocal influence between economic conditions and mating strategies, incorporating cultural, political, and socio-economic contexts. Longitudinal studies examining how shifts in gender equality and economic structures affect homogamy, hypergamous tendencies, and resource acquisition, would further illuminate the interplay between evolutionary and socio-economic factors in shaping modern cyber-mating behaviors.

## Notes

1. For the purposes of the current study our use of the terms “man” and “male” both refer to those who's biological sex was male. Similarly, we use “woman” and “female” interchangeably for those whose biological sex was female. “Sex” to refer to biological differences, particularly where it aligns with the literature on sex-based differences in reproductive strategies and behaviors. “Gender” is used in contexts where participants’ self-identified roles or societal constructs are relevant.

2. Includes participants who indicated that their political views best align with *The Greens (Environmental)* or *Labor Party (Left Wing, Democrat)*.

3. Includes participants who identified their political views as aligning with *Liberal Party (Right Wing, Republican, Conservative)* or *National Party (Country Party, Farmers Party)*.

## Appendix

**Table A1.** Descriptive Statistics for Quasi-Independent Variables.

	*M_Female_*	*M_Male_*	*SD_Female_*	*SD_Male_*	*t*	*p**
Age	38.629	48.723	14.572	14.026	−9.059	.000
Education	5.332	5.102	1.491	1.644	1.755	.080
Earning potential difference	−0.566	0.409	2.306	2.431	−4.431	.000
Capital city	0.685	0.568	0.466	0.496	3.033	.002
Median income	56,742.424	55,240.945	5872.883	6118.358	3.089	.002
Unemployment rate	5.399	5.675	1.882	1.884	−1.832	.067
Sex ratio	98.564	98.546	7.047	5.751	0.036	.972
*Pr (*M_Female_* ≠ *M_Male_*)						

**Table A2.** Descriptive Statistics for Other Control Variables.

	*M_Female_*	*M_Male_*	*SD_Female_*	*SD_Male_*	*t*	*p*
In a relationship	0.345	0.426	0.477	0.495	−2.092	.037
# Children	1.188	1.650	1.385	1.593	−3.333	.001
Frequency of sex (weekly)	2	1.867	2.390	2.274	0.732	.464
Age of ideal partner difference	−0.487	−8.771	6.427	8.227	11.743	.000
Self-rated life satisfaction	72.353	67.420	20.328	24.407	2.591	.010
Self-rated attractiveness	65.032	60.772	18.416	17.609	2.968	.003
Self-rated health	70.930	73.036	17.820	18.331	−1.427	.154
Centralist political views	0.026	0.089	0.159	0.285	−2.699	.007
Right-wing political views	0.160	0.272	0.368	0.445	−2.932	.003
Left-wing political views	0.417	0.274	0.495	0.446	3.554	.000
Non-partisan political views	0.301	0.246	0.460	0.431	1.439	.150
Other political views	0.096	0.119	0.296	0.324	−0.801	.423

*Pr (*M_Female_* ≠ *M_Male_*).

**Table A3.** Non-Linearity in Age for Perceptions of Economic Access.

	Women's economic dependence		Men's ability to find a job	
	Males	Females	Males	Females
*Dependent variable:*	(1)	(2)	(3)	(4)
Age	0.02	1.70*	0.46	−0.51
	(0.04)	(1.69)	(1.15)	(−0.48)
Age # Age	0.00	−0.02	−0.01	0.00
	(0.16)	(−1.53)	(−1.37)	(0.32)
Education	−1.84***	0.86	0.25	1.11
	(−3.56)	(0.52)	(0.45)	(0.66)
Earning potential difference	−0.25	−2.51***	0.35	−1.09
	(−0.64)	(−3.02)	(0.81)	(−1.16)
ln(Income)	−2.00	−82.93***	23.88***	−3.21
	(−0.23)	(−3.32)	(2.75)	(−0.10)
Unemployment rate	−0.38	1.37	0.45	0.68
	(−0.71)	(0.83)	(1.08)	(0.45)
Log difference in income (M-F)	22.51**	−13.12	0.38	−45.57
	(2.07)	(−0.45)	(0.04)	(−1.43)
Difference in unemployment rate (M-F)	0.13	0.82	0.29	2.39**
	(0.33)	(0.72)	(0.97)	(2.01)
Sex ratio (M/F)	−0.06	−0.65*	0.04	0.03
	(−0.44)	(−1.75)	(0.27)	(0.08)
In a relationship	−0.77	−8.34*	2.52	−4.09
	(−0.42)	(−1.71)	(1.43)	(−0.98)
# Children	−0.10	−2.70	1.30*	−0.55
	(−0.16)	(−1.22)	(1.78)	(−0.29)
Frequency of sex (weekly)	−0.21	0.65	0.31	1.11
	(−0.52)	(0.66)	(0.70)	(1.00)
Age of ideal partner difference	0.23*	0.85**	0.09	−0.51
	(1.95)	(2.14)	(0.65)	(−1.14)
Self-rated life satisfaction	−0.03	0.11	0.10**	0.05
	(−0.63)	(0.83)	(2.09)	(0.43)
Self-rated attractiveness	0.02	0.02	0.00	0.12
	(0.27)	(0.12)	(0.04)	(0.80)
Self-rated health	−0.06	0.16	0.02	−0.13
	(−1.00)	(1.33)	(0.39)	(−0.94)
Centralist political views	−2.55	−8.75	−7.93**	22.61***
	(−0.68)	(−0.48)	(−2.32)	(2.64)
Left-wing political views	−6.87***	−10.18	−6.86***	1.11
	(−3.04)	(−1.49)	(−3.07)	(0.18)
Non-partisan political views	−5.61**	−11.69*	−7.75***	4.04
	(−2.19)	(−1.71)	(−3.16)	(0.57)
Other political views	−6.77**	−7.63	−4.28	3.23
	(−2.28)	(−0.85)	(−1.47)	(0.37)
Constant	91.30	962.49***	−229.77**	105.13
	(0.92)	(3.49)	(−2.42)	(0.31)
State FE	Yes	Yes	Yes	Yes
N	680	140	677	140
*Prob. > F*	0.000	0.000	0.000	0.003
*R^2^*	0.079	0.274	0.099	0.147
*R^2^ adj.*	0.041	0.107	0.062	−0.049

*Note.* OLS regressions. Standard errors (in parentheses) are clustered at the postcode level.

**p *< .1; ***p *< .05; ****p *< .01.

**Table A4.** Non-Linearity in Age for Perceptions of Dating Market Access.

	Women's ability to find a date		Men's ability to find a date		Gender difference in dating market access	
	Males	Females	Males	Females	Males	Females
*Dependent variable:*	(1)	(2)	(3)	(4)	(5)	(6)
Age	0.15	2.20**	0.02	−0.37	−0.12	−2.57**
	(0.36)	(2.33)	(0.05)	(−0.36)	(−0.22)	(−2.37)
Age # Age	−0.00	−0.03**	0.00	0.00	0.00	0.03**
	(−1.00)	(−2.41)	(0.04)	(0.06)	(0.77)	(2.17)
Education	−0.05	−0.24	−0.73	−2.18*	−0.68	−1.94
	(−0.08)	(−0.15)	(−1.18)	(−1.74)	(−0.81)	(−1.04)
Earning potential difference	0.79*	0.50	0.82**	−0.25	0.02	−0.72
	(1.82)	(0.50)	(2.01)	(−0.40)	(0.04)	(−0.75)
ln(Income)	11.36	63.45**	24.05**	11.85	12.69	−48.94
	(1.15)	(2.16)	(2.45)	(0.55)	(0.99)	(−1.56)
Unemployment rate	−0.55	3.19**	0.25	2.25**	0.80	−0.91
	(−1.07)	(2.60)	(0.49)	(2.18)	(1.18)	(−0.59)
Log difference in income (M-F)	16.41	−70.25**	−11.64	−7.08	−28.06*	57.45
	(1.35)	(−2.17)	(−0.93)	(−0.27)	(−1.72)	(1.49)
Difference in unemployment rate (M-F)	−0.19	0.33	−0.46	0.83	−0.28	0.39
	(−0.49)	(0.26)	(−1.30)	(0.98)	(−0.55)	(0.27)
Sex ratio (M/F)	0.12	−0.57	−0.09	−0.38	−0.21	0.18
	(0.76)	(−1.13)	(−0.52)	(−1.36)	(−1.00)	(0.30)
In a relationship	−0.48	−1.90	−1.45	1.43	−0.97	3.01
	(−0.23)	(−0.42)	(−0.78)	(0.35)	(−0.37)	(0.56)
# Children	0.36	−2.12	1.79**	−3.37**	1.42	−1.24
	(0.47)	(−1.01)	(2.28)	(−2.39)	(1.52)	(−0.61)
Frequency of sex (weekly)	0.04	0.69	0.86*	−0.31	0.82	−1.05
	(0.07)	(0.64)	(1.83)	(−0.44)	(1.27)	(−1.00)
Age of ideal partner difference	−0.22	0.08	0.14	−0.60	0.36**	−0.69
	(−1.61)	(0.19)	(1.00)	(−1.49)	(2.27)	(−1.49)
Self-rated life satisfaction	−0.02	0.08	−0.00	−0.04	0.02	−0.11
	(−0.40)	(0.62)	(−0.03)	(−0.38)	(0.27)	(−0.78)
Self-rated attractiveness	−0.12*	−0.16	0.09	0.06	0.20**	0.22
	(−1.76)	(−1.11)	(1.29)	(0.60)	(2.19)	(1.46)
Self-rated health	0.11*	0.13	0.09	0.01	−0.02	−0.11
	(1.75)	(0.85)	(1.53)	(0.06)	(−0.19)	(−0.69)
Centralist political views	4.66	12.07	2.78	34.01***	−1.87	21.62*
	(1.15)	(0.78)	(0.72)	(3.60)	(−0.35)	(1.93)
Left-wing political views	0.50	11.88*	−1.53	18.68***	−2.02	7.19
	(0.19)	(1.95)	(−0.65)	(3.02)	(−0.58)	(0.94)
Non-partisan political views	−2.37	11.39	−5.71**	19.24***	−3.34	7.87
	(−0.88)	(1.55)	(−2.14)	(2.86)	(−0.89)	(0.80)
Other political views	−2.50	17.61*	1.23	13.28	3.73	−4.16
	(−0.71)	(1.79)	(0.40)	(1.58)	(0.87)	(−0.36)
Constant	−72.89	−629.96*	−218.09**	−29.22	−145.27	573.74*
	(−0.68)	(−1.96)	(−2.02)	(−0.12)	(−1.01)	(1.70)
State FE	Yes	Yes	Yes	Yes	Yes	Yes
N	673	139	674	140	673	139
*Prob. > F*	0.007	0.000	0.000	0.000	0.000	0.026
*R^2^*	0.053	0.224	0.088	0.323	0.075	0.215
*R^2^ adj.*	0.013	0.043	0.050	0.167	0.036	0.033

*Note.* OLS regressions. Standard errors (in parentheses) are clustered at the postcode level.

**p *< .1; ***p *< .05; ****p *< .01.

**Table A5.** SOI-R Robustness Check for Perceptions of Dating Market Access.

	Women's ability to find a date		Men's ability to find a date		Gender difference in dating market access	
	Males	Females	Males	Males	Females	Males
*Dependent variable:*	(1)	(2)	(3)	(1)	(2)	(3)
Age	−0.26***	−0.14	0.04	−0.37	0.29**	−0.24
	(−2.76)	(−0.51)	(0.43)	(−1.53)	(2.50)	(−0.69)
Education	−0.13	0.04	−0.74	−2.16*	−0.61	−2.20
	(−0.21)	(0.03)	(−1.20)	(−1.78)	(−0.73)	(−1.15)
Earning potential difference	0.70	0.58	0.81**	−0.15	0.10	−0.70
	(1.60)	(0.62)	(1.99)	(−0.23)	(0.18)	(−0.74)
ln(Income)	10.51	45.91	24.01**	3.17	13.51	−40.12
	(1.07)	(1.48)	(2.46)	(0.13)	(1.06)	(−1.13)
Unemployment rate	−0.50	3.51***	0.25	2.30**	0.75	−1.17
	(−0.96)	(2.89)	(0.50)	(2.23)	(1.12)	(−0.77)
Log difference in income (M-F)	16.40	−58.59*	−11.91	−2.50	−28.33*	50.23
	(1.38)	(−1.68)	(−0.96)	(−0.10)	(−1.78)	(1.18)
Difference in unemployment rate (M-F)	−0.25	0.61	−0.47	0.81	−0.23	0.08
	(−0.64)	(0.49)	(−1.33)	(1.01)	(−0.46)	(0.06)
Sex ratio (M/F)	0.12	−0.50	−0.09	−0.40	−0.21	0.09
	(0.76)	(−1.07)	(−0.53)	(−1.39)	(−1.02)	(0.16)
In a relationship	−0.32	2.06	−1.48	3.42	−1.16	1.06
	(−0.16)	(0.38)	(−0.80)	(0.76)	(−0.46)	(0.16)
# Children	0.33	−1.76	1.78**	−3.59**	1.45	−1.82
	(0.43)	(−0.85)	(2.27)	(−2.60)	(1.54)	(−0.88)
Frequency of sex (weekly)	−0.17	0.17	0.81*	−0.58	0.99	−0.81
	(−0.35)	(0.17)	(1.67)	(−0.92)	(1.50)	(−0.80)
SOI-R	0.65***	1.26**	0.11	0.62	−0.54*	−0.63
	(2.77)	(2.36)	(0.49)	(1.26)	(−1.78)	(−0.93)
Age of ideal partner difference	−0.16	0.27	0.15	−0.57	0.31*	−0.85*
	(−1.15)	(0.65)	(1.08)	(−1.54)	(1.91)	(−1.74)
Self-rated life satisfaction	−0.01	0.05	0.00	−0.05	0.01	−0.09
	(−0.26)	(0.42)	(0.00)	(−0.50)	(0.19)	(−0.64)
Self-rated attractiveness	−0.14**	−0.15	0.08	0.04	0.23**	0.19
	(−2.19)	(−1.03)	(1.20)	(0.41)	(2.45)	(1.28)
Self-rated health	0.12*	0.15	0.10	0.03	−0.02	−0.11
	(1.80)	(1.04)	(1.55)	(0.26)	(−0.24)	(−0.66)
Centralist political views	4.61	7.80	2.77	32.96***	−1.84	24.80*
	(1.16)	(0.49)	(0.72)	(3.76)	(−0.34)	(1.85)
Left-wing political views	0.43	11.58*	−1.57	18.71***	−2.00	7.52
	(0.17)	(1.84)	(−0.67)	(3.06)	(−0.58)	(0.94)
Non-partisan political views	−2.37	12.54*	−5.75**	19.09***	−3.37	6.58
	(−0.89)	(1.68)	(−2.16)	(2.95)	(−0.91)	(0.65)
Other political views	−2.72	17.31*	1.13	12.77	3.85	−4.37
	(−0.79)	(1.83)	(0.37)	(1.58)	(0.92)	(−0.37)
Constant	−63.27	−423.82	−219.16**	57.84	−155.95	455.07
	(−0.59)	(−1.25)	(−2.05)	(0.23)	(−1.10)	(1.20)
State FE	Yes	Yes	Yes	Yes	Yes	Yes
N	673	139	674	140	673	139
*Prob. > F*	0.000	0.000	0.000	0.000	0.000	0.085
*R^2^*	0.063	0.238	0.089	0.338	0.078	0.198
*R^2^ adj.*	0.024	0.061	0.051	0.186	0.040	0.011

*Note.* OLS regressions. Standard errors (in parentheses) are clustered at the postcode level.

**p *< .1; ***p *< .05; ****p *< .01.

## References

[bibr1-14747049241310154] ABS Geospatial Solutions. (2016). *ASGS geographic correspondences (2016)*. Retrieved August 16, 2022, from https://data.gov.au/data/dataset/asgs-geographic-correspondences-2016

[bibr2-14747049241310154] Australian Bureau of Statistics. (2018–19). ‘Table 4: Employee income by age and sex 2014–15 to 2018–19’ *Personal income in Australia*. Retrieved August 16, 2022, from https://www.abs.gov.au/statistics/labour/earnings-and-working-conditions/personal-income-australia/2014-15-2018-19

[bibr3-14747049241310154] Australian Bureau of Statistics. (2019). *Regional population by age and sex, Australia, 2019 (3235.0)*. Australian Government. Retrieved August 16, 2022, from https://data.aurin.org.au/dataset/au-govt-abs-abs-regional-population-summary-sa2-2019-sa2-2016

[bibr4-14747049241310154] Australian Bureau of Statistics. (2019). *Share of state population change*. https://www.abs.gov.au/articles/50-years-capital-city-population-change

[bibr5-14747049241310154] Autor D. Dorn D. Hanson G. (2019). When work disappears: Manufacturing decline and the falling marriage market value of young men. American Economic Review: Insights, 1(2), 161–178. 10.1257/aeri.20180010

[bibr6-14747049241310154] Baele S. J. Brace L. Coan T. G. (2021). From “incel” to “saint”: Analyzing the violent worldview behind the 2018 Toronto attack. Terrorism and Political Violence, 33(8), 1667–1691. 10.1080/09546553.2019.1638256

[bibr7-14747049241310154] Barber N. (2001). On the relationship between marital opportunity and teen pregnancy: The sex ratio question. Journal of Cross-Cultural Psychology, 32(3), 259–267. 10.1177/0022022101032003001

[bibr8-14747049241310154] Baumeister R. F. (2000). Gender differences in erotic plasticity: The female sex drive as socially flexible and responsive. Psychological Bulletin, 126(3), 347–374. 10.1037/0033-2909.126.3.347 10825779

[bibr9-14747049241310154] Baumeister R. F. Reynolds T. Winegard B. Vohs K. D. (2017). Competing for love: Applying sexual economics theory to mating contests. Journal of Economic Psychology, 63, 230–241. 10.1016/j.joep.2017.07.009

[bibr10-14747049241310154] Baumeister R. F. Twenge J. M. (2002). Cultural suppression of female sexuality. Review of General Psychology, 6(2), 166–203. 10.1037/1089-2680.6.2.166

[bibr11-14747049241310154] Baumeister R. F. Vohs K. D. (2004). Sexual economics: Sex as female resource for social exchange in heterosexual interactions. Personality and Social Psychology Review, 8(4), 339–363. 10.1207/s15327957pspr0804_2 15582858

[bibr12-14747049241310154] Becker G. S. (1973). A theory of marriage: Part I. Journal of Political Economy, 81(4), 813–846. 10.1086/260084

[bibr13-14747049241310154] Becker G. S. Lewis H. G. (1973). On the interaction between the quantity and quality of children. Journal of Political Economy, 81(2, Part 2), S279–S288. 10.1086/260166

[bibr14-14747049241310154] Betzig L. L. (1986). Despotism and differential reproduction: A Darwinian view of history. Aldine Publishing Co.

[bibr15-14747049241310154] Betzig L. (1993). Sex, succession, and stratification in the first six civilizations: How powerful men reproduced, passed power on to their sons, and used power to defend their wealth, women, and children. In Ellis L. (Ed.), Social stratification and socioeconomic inequality: Vol. 1. A comparative biosocial analysis (pp. 37–74). Praeger Publishers/Greenwood Publishing Group.

[bibr16-14747049241310154] Blake K. R. Bastian B. Denson T. F. Grosjean P. Brooks R. C. (2018). Income inequality not gender inequality positively covaries with female sexualization on social media. Proceedings of the National Academy of Sciences, 115(35), 8722–8727. 10.1073/pnas.1717959115 PMC6126749 30131431

[bibr17-14747049241310154] Blake K. Brooks R. C. (2019). Income inequality and reproductive competition: Implications for consumption, status-seeking, and women’s self-sexualization. The Social Psychology of Inequality, 173–185. 10.1007/978-3-030-28856-3_11

[bibr18-14747049241310154] Blake K. Godwin M. Whyte S. (2020). “I sexually identify as an Attack Helicopter”: Incels, trolls, and non-binary gender politics online . *First Monday* .

[bibr19-14747049241310154] Blau F. D. Kahn L. M. Waldfogel J. (2000). Understanding young women’s marriage decisions: The role of labor and marriage market conditions. ILR Review, 53(4), 624–647. 10.1177/001979390005300404

[bibr20-14747049241310154] Brickell C. (2006). The sociological construction of gender and sexuality. The Sociological Review, 54(1), 87–113. 10.1111/j.1467-954X.2006.00603.x

[bibr21-14747049241310154] Brooks R. C. Blake K. R. Fromhage L. (2022). Effects of gender inequality and wealth inequality on within-sex mating competition under hypergyny. Evolution and Human Behavior, 43(6), 501–509. 10.1016/j.evolhumbehav.2022.08.006

[bibr22-14747049241310154] Brooks R. C. Russo-Batterham D. Blake K. R. (2022). Incel activity on social media linked to local mating ecology. Psychological Science, 33(2), 249–258. 10.1177/09567976211036065 35015599

[bibr23-14747049241310154] Brooks R. Scott I. M. Maklakov A. A. Kasumovic M. M. Clark A. P. Penton-Voak I. S. (2011). National income inequality predicts women's preferences for masculinized faces better than health does. Proceedings of the Royal Society B: Biological Sciences, 278(1707), 810–812. 10.1098/rspb.2010.0964 PMC3049041 21147809

[bibr24-14747049241310154] Buss D. M. (1985). Human mate selection: Opposites are sometimes said to attract, but in fact we are likely to marry someone who is similar to us in almost every variable. American Scientist, 73(1), 47–51. https://www.jstor.org/stable/27853061

[bibr25-14747049241310154] Buss D. M. (1989). Sex differences in human mate preferences: Evolutionary hypotheses tested in 37 cultures. Behavioral and Brain Sciences, 12(1), 1–14. 10.1017/S0140525X00023992

[bibr26-14747049241310154] Buss D. M. Schmitt D. P. (1993). Sexual strategies theory: An evolutionary perspective on human mating. Psychological Review, 100(2), 204–232. 10.1037/0033-295X.100.2.204 8483982

[bibr27-14747049241310154] Chan H. F. Torgler B. Whyte S. (2021). Exploring sexual orientation beyond genital arousal: Using large-scale online dating contact behavior to study male and female bisexuality. Proceedings of the National Academy of Sciences, 118(12), e2026320118. 10.1073/pnas.2026320118 PMC8000017 33723079

[bibr28-14747049241310154] DeLamater J. D. Hyde J. S. (1998). Essentialism vs. Social constructionism in the study of human sexuality. The Journal of Sex Research, 35(1), 10–18. 10.1080/00224499809551913

[bibr29-14747049241310154] Dickemann M. (1979). The ecology of mating systems in hypergynous dowry societies. Social Science Information, 18(2), 163–195. 10.1177/053901847901800201

[bibr30-14747049241310154] Farrell T. Fernandez M. Novotny J. Alani H. (2019, June). Exploring misogyny across the manosphere in Reddit. In Proceedings of the 10th ACM Conference on Web Science, 87–96. 10.1145/3292522.3326045

[bibr31-14747049241310154] Fiore A. T. Taylor L. S. Zhong X. Mendelsohn G. A. Cheshire C. (2010, January). Who's right and who writes: People, profiles, contacts, and replies in online dating. In 2010 43rd Hawaii International Conference on System Sciences (pp. 1–10). IEEE.

[bibr32-14747049241310154] Gangestad S. W. Simpson J. A. (2000). The evolution of human mating: Trade-offs and strategic pluralism. Behavioral and Brain Sciences, 23(4), 573–587. 10.1017/S0140525X0000337X 11301543

[bibr33-14747049241310154] Hitsch G. J. Hortaçsu A. Ariely D. (2010). What makes you click?—Mate preferences in online dating. Quantitative Marketing and Economics, 8(4), 393–427. 10.1007/s11129-010-9088-6

[bibr34-14747049241310154] Hopcroft R. L. (2006). Sex, status, and reproductive success in the contemporary United States. Evolution and Human Behavior, 27(2), 104–120. 10.1016/j.evolhumbehav.2005.07.004

[bibr35-14747049241310154] Hopcroft R. L. (2021). High income men have high value as long-term mates in the U.S.: Personal income and the probability of marriage, divorce, and childbearing in the U.S. Evolution and Human Behavior, 42(5), 409–417. 10.1016/j.evolhumbehav.2021.03.004

[bibr36-14747049241310154] Kennair L. E. O. Thomas A. G. Buss D. M. Bendixen M. (2023). Examining the sexual double standards and hypocrisy in partner suitability appraisals within a Norwegian sample. Evolutionary Psychology, 21(1), 14747049231165687. 10.1177/14747049231165687 36972495 PMC10303487

[bibr37-14747049241310154] Lee L. Loewenstein G. Ariely D. Hong J. Young J. (2008). If I'm not hot, are you hot or not? Physical-attractiveness evaluations and dating preferences as a function of one's own attractiveness. Psychological Science, 19(7), 669–677. 10.1111/j.1467-9280.2008.02141.x 18727782

[bibr38-14747049241310154] Loughran D. S. (2002). The effect of male wage inequality on female age at first marriage. Review of Economics and Statistics, 84(2), 237–250. 10.1162/003465302317411505

[bibr39-14747049241310154] Nettle D. Pollet T. V. (2008). Natural selection on male wealth in humans. The American Naturalist, 172(5), 658–666. 10.1086/591690 18808363

[bibr40-14747049241310154] Penke L. Asendorpf J. B. (2008). Beyond global sociosexual orientations: A more differentiated look at sociosexuality and its effects on courtship and romantic relationships. Journal of Personality and Social Psychology, 95(5), 1113–1135. 10.1037/0022-3514.95.5.1113 18954197

[bibr41-14747049241310154] Price M. E. Pound N. Scott I. M. (2014). Female economic dependence and the morality of promiscuity. Archives of Sexual Behavior, 43(7), 1289–1301. 10.1007/s10508-014-0320-4 24961579 PMC4161927

[bibr42-14747049241310154] Relationships Australia. (2023) Retrieved January 5, 2023, from: https://relationships.org.au/document/november-2017-online-dating/

[bibr43-14747049241310154] Schmitt D. P. (2005). Sociosexuality from Argentina to Zimbabwe: A 48-nation study of sex, culture, and strategies of human mating. Behavioral and Brain Sciences, 28(2), 247–275. 10.1017/S0140525X05000051 16201459

[bibr44-14747049241310154] Skopek J. Schulz F. Blossfeld H. P. (2011). Who contacts whom? Educational homophily in online mate selection. European Sociological Review, 27(2), 180–195. 10.1093/esr/jcp068

[bibr45-14747049241310154] Taylor S. D. Charlton J. P. Ranyard R. (2012). Ethnic and gender differences in the labour market perceptions of post-higher education job seekers: ‘double jeopardy’ or ‘ethnic prominence’? Journal of Occupational and Organizational Psychology, 85(2), 353–369. 10.1111/j.2044-8325.2011.02041.x

[bibr46-14747049241310154] Van Der Klaauw W. (1996). Female labour supply and marital Status decisions: A life-cycle model. The Review of Economic Studies, 63(2), 199–235. 10.2307/2297850

[bibr47-14747049241310154] Van Leeuwen M. H. Maas I. (2010). Historical studies of social mobility and stratification. Annual Review of Sociology, 36(1), 429–451. 10.1146/annurev.soc.012809.102635

[bibr48-14747049241310154] Walter K. V. Conroy-Beam D. Buss D. M. Asao K. Sorokowska A. Sorokowski P. Aavik T. Akello G. Alhabahba M. M. Alm C. Amjad N. Anjum A. Atama C. S. Duyar D. A. Ayebare R. Batres C. Bendixen M. Bensafia A. Bizumic B. Boussena M. Butovskaya M. Can S. Zupančič M. (2020). Sex differences in mate preferences across 45 countries: A large-scale replication. Psychological Science, 31(4), 408–423. 10.1177/0956797620904154 32196435

[bibr49-14747049241310154] Whyte S. Brooks R. C. Chan H. F. Torgler B. (2021). Sex differences in sexual attraction for aesthetics, resources and personality across age. PloS One, 16(5), e0250151. 10.1371/journal.pone.0250151 PMC8133465 34010298

[bibr50-14747049241310154] Whyte S. Brooks R. C. Torgler B. (2018). Man, woman, “other”: Factors associated with nonbinary gender identification. Archives of Sexual Behavior, 47(8), 2397–2406. 10.1007/s10508-018-1307-3 30255409

[bibr51-14747049241310154] Whyte S. Brooks R. C. Torgler B. (2019). Sexual economic theory & the human mating market. Applied Economics, 51(57), 6100–6112. 10.1080/00036846.2019.1650886

[bibr52-14747049241310154] Whyte S. Chan H. F. Torgler B. (2018). Do men and women know what they want? Sex differences in online daters’ educational preferences. Psychological Science, 29(8), 1370–1375. 10.1177/0956797618771081 29932820

[bibr53-14747049241310154] Whyte S. Savage D. A. Torgler B. (2017). Online sperm donors: The impact of family, friends, personality and risk perception on behaviour. Reproductive Biomedicine Online, 35(6), 723–732. 10.1016/j.rbmo.2017.08.023 28951001

[bibr54-14747049241310154] Whyte S. Torgler B. (2016). Determinants of online sperm donor success: How women choose. Applied Economics Letters, 23(8), 592–596. 10.1080/13504851.2015.1090543

[bibr55-14747049241310154] Whyte S. Torgler B. (2017). Preference versus choice in online dating. Cyberpsychology, Behavior and Social Networking, 20(3), 150–156. 10.1089/cyber.2016.0528 28263677

[bibr56-14747049241310154] Wiederman M. W. (2001). Understanding sexuality research. Wadsworth Publishing Company.

[bibr57-14747049241310154] Wiederman M. W. Allgeier E. R. (1992). Gender differences in mate selection criteria: Sociobiological or socioeconomic explanation? Ethology & Sociobiology, 13(2), 115–124. 10.1016/0162-3095(92)90021-U

[bibr58-14747049241310154] Wooding S. Ostler C. Prasad B. R. Watkins W. S. Sung S. Bamshad M. Jorde L. B. (2004). Directional migration in the Hindu castes: Inferences from mitochondrial, autosomal and Y-chromosomal data. Human Genetics, 115(3), 221–229. 10.1007/s00439-004-1130-x 15232732

